# Nutritional factors associated with femoral neck bone mineral density in children and adolescents

**DOI:** 10.1186/s12891-019-2901-9

**Published:** 2019-11-07

**Authors:** Guo-Hau Gou, Feng-Jen Tseng, Sheng-Hao Wang, Pao-Ju Chen, Jia-Fwu Shyu, Ru-Yu Pan

**Affiliations:** 1 Graduate Institute of Medical Sciences, National Defense Medical Center, No. 161, Sec.6 Minquan E. Rd., Neihu Dist., Taipei, 11490 Taiwan, Republic of China; 2 Department of Nursing, Hsin Sheng Junior College of Medical Care and Management, Taoyuan, 32544 Taiwan, Republic of China; 3 Department of Orthopedics, Hualien Armed Force Hospital, Hualien, 971, Taiwan, Republic of China; 4 Department of Life Science and the Institute of Biotechnology, National Dong Hwa University, Hualien, 974, Taiwan, Republic of China; 5 Department of Orthopaedics, Tri-Service General Hospital, National Defense Medical Center, Taipei, 11490 Taiwan, Republic of China; 6 Department of Biology and Anatomy, National Defense Medical Center, Taipei, 11490 Taiwan, Republic of China

**Keywords:** Macronutrients, Micronutrients, Femoral bone density, Children, Adolescent, NHANES

## Abstract

**Background:**

Nutritional factors including vitamin D, magnesium, and fat are known to affect bone mineral accrual. This study aimed to evaluate associations between dietary nutrient intakes (both macronutrients and micronutrients) and bone mineral density (BMD) in children and adolescents.

**Methods:**

Data for this cross-sectional, population-based study were derived from the National Health and Nutrition Examination Survey (NHANES). Participants aged from 8 to 19 years were included. The primary outcome was femoral neck BMD.

**Results:**

Multivariate analyses revealed that for participants aged 8 to 11, daily sodium intake was significantly and positively associated with femoral neck BMD (B = 0.9 ×  10^− 5^, *p* = 0.031); in particular, subgroup analyses by sex found that in male participants aged 8–11, daily total cholesterol intake (B = 5.3 × 10^− 5^, *p* = 0.030) and calcium intake (B = − 2.0 × 10^− 5^, *p* < 0.05) were significantly associated with femoral neck BMD in a positive and negative manner, respectively, but neither were observed in female participants of this age group. In contrast, daily intakes of vitamin D and magnesium were significantly and positively associated with femoral neck BMD in female participants aged 8–11 (B = 246.8 × 10^− 5^ and 16.3 × 10^− 5^, *p* = 0.017 and 0.033, respectively). For participants aged 16 to 19, daily total fat intake was significantly and negatively associated with femoral neck BMD (B = − 58 × 10^− 5^, *p* = 0.048); further stratification by sex found that magnesium and sodium intakes were significantly and positively associated with femoral neck BMD only in females of this age group (B = 26.9 × 10^− 5^ and 2.1 × 10^− 5^, respectively; both *p* < 0.05). However, no significant associations between daily nutrient intakes and femoral neck BMD were identified in participants aged 12–15 before or after subgroup stratification.

**Conclusion:**

The study found that associations of specific nutrition-related variables with BMD of the femoral neck is dependent upon age and gender.

## Background

Peak bone mass is an important determinant for the lifetime risk of osteoporosis, which in turn can increase the susceptibility to fracture and associated morbidity and mortality in adulthood [1]. Childhood and adolescence are critical periods of life for bone development and bone mineralization, which then contribute to bone mass accumulation that typically peaks between 16 and 24 years of age [2]. Puberty is a time of major differences between sexs in bone growth, particularly with respect to bone mass content and bone size [3]. The bone mineral density (BMD) of the femoral neck has been used by several groups as an indicator of development and health in children [4–7]. BMD and bone mineral content of the femoral neck was shown first to reach plateau compared to those of the lumber spine and the whole body [6]. Furthermore, gender is also considered an issue in the timing of and perhaps contributing factors to bone mass accrual in the early stages of life [4, 6]. Femoral BMD correlated with osteopenia and osteoporosis in school children aged between 8 and 18 years, and especially in girls [4]. In boys, weight, lean body mass and body-mass-index (BMI) were found positively associated with bone mineral apparent density of the lumbar spine and femoral neck, rather than with whole body and the radius [5]. Bone mineral parameters of the femoral neck also showed significant positive associations with physical activity in adolescent boys [7].

Approximately 60 to 80% of peak bone mass variance is dependent upon genetic factors, other determinants include modifiable factors such as diet and exercise [8]. Nutrition factors that are well known to affect bone mineral accrual are calcium and vitamin D [9, 10]. These nutrients are constituents of bone and are biologically relevant for the growth and mineralization of the bone. Several other nutritional factors are also thought to be important in bone growth and mineralization, such as magnesium, phosphorous, potassium, fatty acids, protein vitamin C, and vitamin K; however, the scientific evidence of their importance is limited [11]. Nutrition may influence bone strength through several mechanisms such as impacting longitudinal growth, bone stiffness, and muscle strength which is necessary to put strain on the bone for proper bone development [12]. Calcium and vitamin D intakes were associated positively with femoral neck BMD in young men (16–24 years-old) in Cananda, [13] while a different study further indicated this positive association in school children and adolescents (8–18 years old) [4]. On the other hand, diets rich in dark green vegetables, eggs, fruits, whole grains, and nuts are associated with bone health indicators including femoral neck BMD in adolescents and adults [14, 15].

However, the potential contributions of micronutrients and macronutrients to bone health have rarely been compared in older children, children during puberty, and adolescents in the same study. Hence, the purpose of the present cross-sectional study was to evaluate the potential contributions of macronutrients and micronutrients on femoral neck BMD in children and adolescents using data derived from a population-based database.

## Methods

### Data source

Data for this cross-sectional study were derived from the National Health and Nutrition Examination Survey (NHANES), which is administered by the National Center for Health Statistics of the Centers for Disease Control and Prevention (https://www.cdc.gov/Nchs/Nhanes/about_nhanes.htm). The NHANES data represent the national, non-institutionalized population of the United States.

### Study population

Data from 2 cycles of NHANES during 2007–2010 were used, and the participants aged from 8 to 19 years were included. The exclusion criteria were i) participants without femoral neck BMD data, or body weight data, ii) participants with extreme obesity defined as those with BMI above 120% of the upper 95th percentile of sex- and age-matched data, which was derived from the 2000 CDC growth charts and a CDC SAS program, and iii) participants with lower energy intake (< 400 Kcal) or higher energy intake (> 3000 Kcal). The participants with extreme obesity or implausible energy intakes were excluded due to the possible bias of diet. The study population was stratified by age and sex to evaluate the associations between study variables and femoral neck BMD.

### Primary outcome and study variables

The primary outcome was femoral neck BMD. The study variables were daily intakes of individual macronutrients and micronutrients (from both dietary intake and supplement). Macronutrients of interest included energy, protein, carbohydrate, total sugars, dietary fiber, total fat, total saturated fatty acids, and cholesterol. Micronutrients included vitamins (vitamin D, vitamin K, and vitamin C) and minerals (calcium, magnesium, and sodium). Participants’ profiles included demographics (age, race, sex, body weight, body height, body mass index, and family income), physical activity (vigorous and moderate recreational activities based on NHANES questionnaire addressing the type and duration of sporting activity), and soft drink availability at home. Low family income is defined as a ratio of family income to poverty < 3.0; a ratio ≥ 3.0 is defined as high family income.

### Statistical analysis

For the study population descriptive statistics were summarized after stratification by three age groups. Unweighted counts and weighted proportion are presented for categorical variables sex, race, income, physical activity and at home soft drink availability. Weighted means and standard errors (SE) were provided for continuous variables. Weights used to provide national estimates were provided by NHANES. The association of daily nutritional intake with femoral neck BMD were evaluated by univariate and multivariate general linear models in participants aged 8 to 11, 12 to 15, and 16 to 19. In order to minimize the effects of individual differences in growth rate across children (or adolescents) of the same age and factors known to affect body development [16, 17], multivariate models were adjusted for race, sex, body weight, body height, family income, physical activity, soft drink availability, and total energy intake. NHANES does not collect information on physical exercise for participants younger than 12; therefore, multivariate models for participants aged between 8 and 11 were not adjusted for physical activity. Multivariate regression models for each age group were also analyzed after further stratifying by sex. A two-tailed *P*-value less than 0.05 was considered significant. All statistical analyses were carried out with IBM SPSS Statistics for Windows, Version 22.0. (IBM Corp., Armonk, NY, USA).

## Results

### Study population

**Fig. 1:**
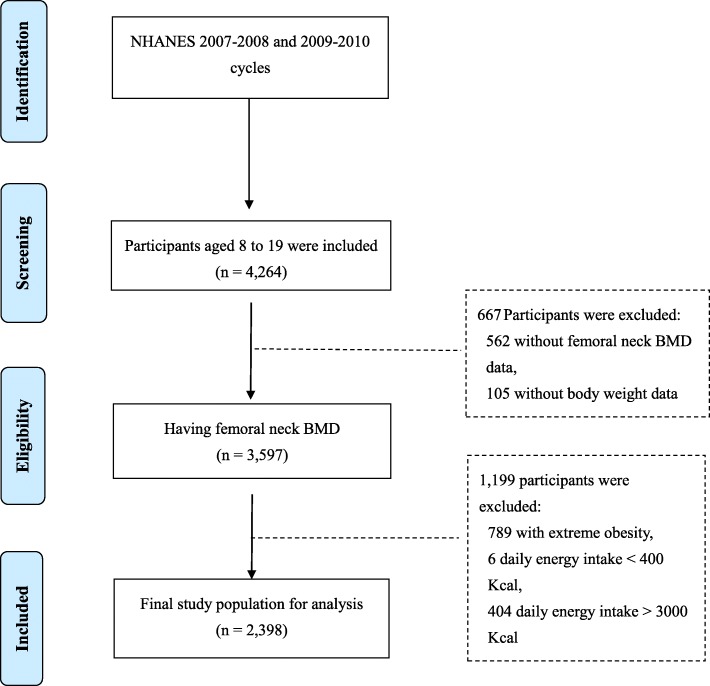
Flowchart for inclusion and exclusion criteria

A total of 4264 child and adolescent participants (aged 8–19) were initially extracted from the NHANES 2007–2008 and 2009–2010 cycles. Of them, participants who had no data for femoral neck BMD (*n* = 562) or had missing NHANES sample weights (*n* = 105) were excluded. In addition, 789 participants with extreme obesity and 410 participants with implausible energy intakes (< 400 kcal or > 3000 kcal) were also excluded. As a result, the final study population included 2398 child and adolescent participants (Fig. 1). The included participants were then divided into three age groups: aged 8–11, aged 12–15, and aged 16–19 with the mean femoral neck BMD of 0.709, 0.921, and 1.015, respectively (Table 1).

**Table 1:** Descriptive statistics for demographics, femoral neck BMD, physical activity, and soft drink availability at home among the three age groups

Variables	Age groups, yrs		
	8–11 (*n* = 1004)	12–15 (*n* = 725)	16–19 (*n* = 669)
Demographics			
Race, Non-Hispanic White (%)	329 (59.6)	232 (60.3)	224 (61.8)
Sex, Males (%)	479 (48.0)	358 (47.2)	1163 (46.5)
Body weight (mean ± SE, kg)	34.6 ± 0.3	54.9 ± 0.6	64.4 ± 0.6
Body height (mean ± SE, cm)	139.5 ± 0.4	162.6 ± 0.4	168.9 ± 0.5
Body mass index (mean ± SE, kg/m^2^)	17.5 ± 0.1	20.6 ± 0.2	22.5 ± 0.1
Income, Low-income^a^	694 (55.4)	494 (55.5)	412 (49.9)
Femoral neck bone mineral density (BMD) (mean ± SE, g/cm^2^)	0.709 ± 0.004	0.921 ± 0.004	1.015 ± 0.006
Physical activity^b^			
Vigorous recreational activities, yes (%)	–	488 (70.3)	372 (57.1)
Moderate recreational activities, yes (%)	–	380 (59.6)	295 (47.9)
Soft drink availability at home^c^			
Always	361 (38.3)	280 (37.4)	278 (42.6)
Most of the time	149 (16)	105 (14.5)	96 (14.1)
Sometimes	236 (21.3)	182 (22.2)	140 (15.5)
Rarely	161 (14.9)	112 (17.2)	81 (16.3)
Never	89 (8.8)	41 (7.9)	63 (9.6)

^a^ Low income: participants with a ratio of family income to poverty < 3.0; otherwise rate ≥ 3.0 indicated high income

^b^ The definition of vigorous and moderate recreational activity was based on the NHANES questionnaire (https://wwwn.cdc.gov/Nchs/Nhanes/2013-2014/PAQ_H.htm) with indicated types and duration of sports

^c^ 24 participants with missing record were not presented

**Fig. 2:**
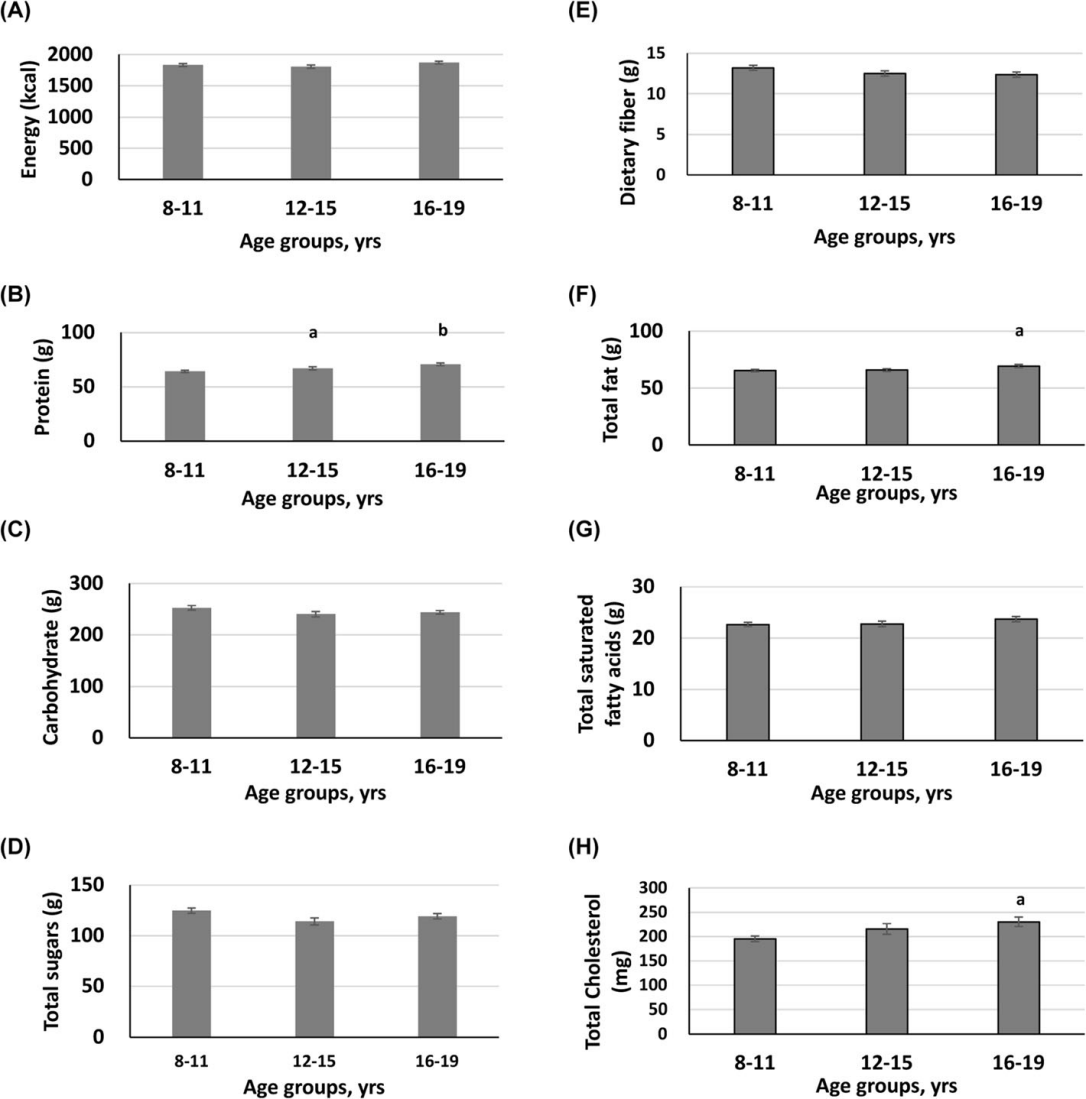
Descriptive statistics for daily macronutrient intakes for different age groups. Data are presented as mean ± SE. ^a, b^*p* < 0.05, significant differences as compared to participants aged 8-11^a^ or 12-15^b^

**Fig. 3:**
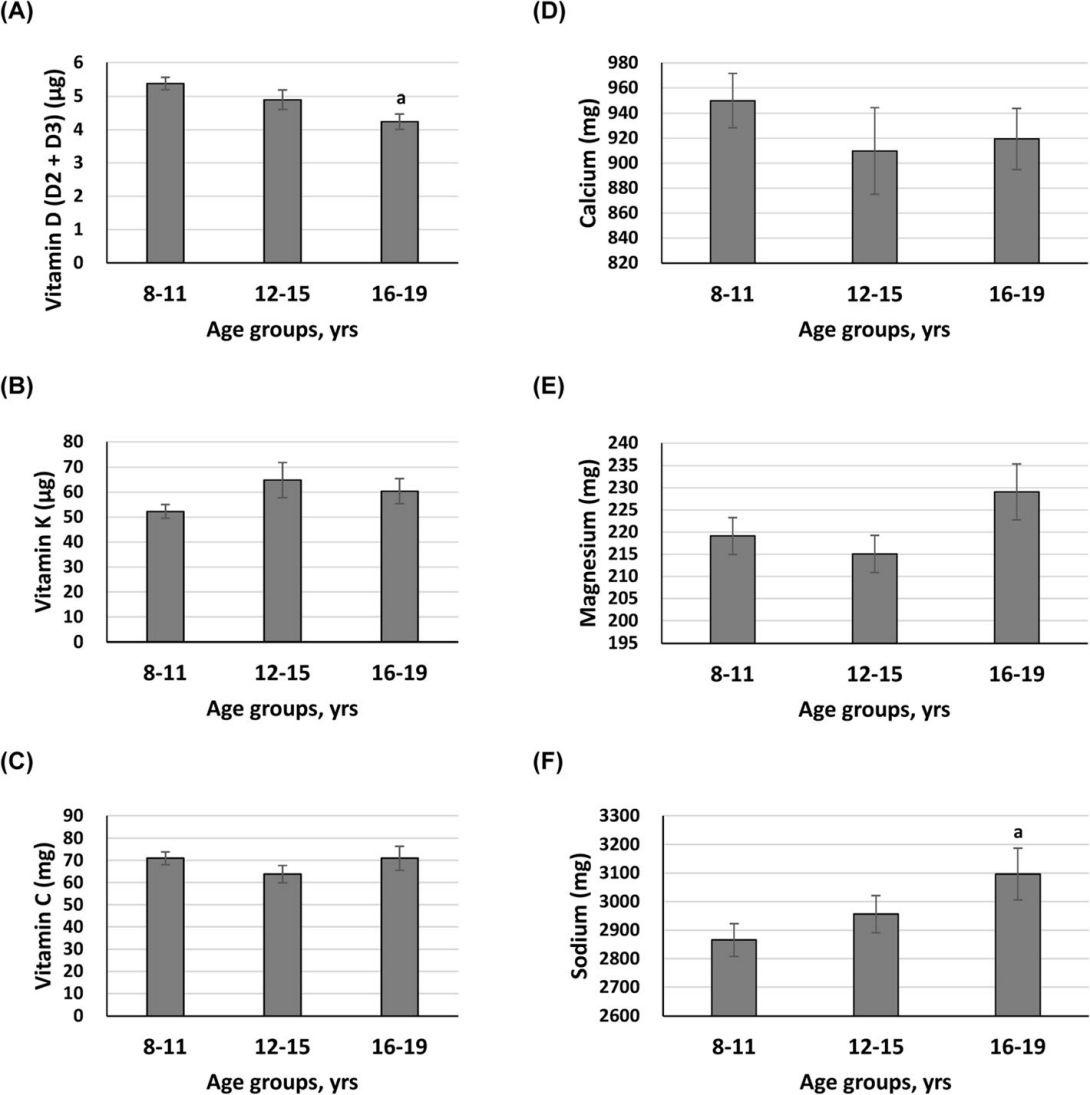
Descriptive statistics for daily micronutrient intakes for different age groups. Data were presented as mean ± SE. ^a^*p* < 0.05, significant difference as compared to participants aged 8–11. No statistically significant differences were observed between 12 and 15 and the other two groups

Comparisons of daily intakes of macronutrients and micronutrients between age groups are shown in Figs. 2 and 3, respectively. Among daily macronutrient intakes, daily protein intake significantly increased with age among three age groups (Fig. 2). Daily intakes of total fat and total cholesterol were significantly higher in participants aged 16–19, compared to those of participants aged 8–11. However, daily intakes of energy, carbohydrate, total sugar, dietary fiber, and total saturated fatty acid were comparable among three age groups. Regarding daily micronutrient intakes, compared to participants aged 8–11, participants aged 16–19 had significantly lower daily vitamin D intake but significantly higher daily sodium intake (Fig. 3). No significant differences in daily intakes of vitamin K, vitamin D, calcium, and magnesium were observed among the three age groups (Fig. 3).

### The associations between femoral neck BMD and intake of macronutrients and micronutrients across different age groups

#### Ages 8 to 11 years

Univariate analyses revealed that daily intakes for energy, protein, and total fat were significantly and positively associated with femoral neck BMD in participants aged 8 to 11 (B = 1.2 × 10^− 5^, 30.4 × 10^− 5^, and 24.7 × 10^− 5^, and *p* = 0.018, 0.049, and 0.029, respectively) (Additional file 1: Table S1). Regarding micronutrients, univariate analyses revealed that daily intakes of magnesium and sodium (B = 8 × 10^− 5^ and 0.9 × 10^− 5^, *p* = 0.040 and *p* < 0.001, respectively), but not daily vitamin or calcium intakes, were significantly and positively associated with femoral neck BMD in participants aged 8 to 11 (Additional file 1: Table S1).

After adjusting for race, sex, body weight, body height, family income, soft drink availability, physical activity, and energy, multivariate analyses indicated that daily macronutrient intakes were not associated with femoral neck BMD in participants aged 8 to 11 (Table 2). Meanwhile, multivariate analyses found that daily sodium intake was significantly and positively associated with femoral neck BMD (B = 0.9 × 10^− 5^, *p* = 0.031) (Table 3).

**Table 2:** ^a^ Multivariate analyses of associations between daily macronutrient intakes and femoral neck BMD in three age groups

	Ages 8 to 11 (*n* = 1004)		Ages 12 to 15 (*n* = 725)		Ages 16 to 19 (*n* = 669)	
	B × 10^− 5^ (95% CI)	*p*-value	B × 10^− 5^ (95% CI)	*p*-value	B × 10^− 5^ (95% CI)	*p*-value
Daily total nutrition intake						
Energy (kcal)	Not applicable		Not applicable		Not applicable	
Protein (g)	15.7 (−30.8, 62.2)	0.498	−21.1 (− 67.6, 25.3)	0.361	23.2 (− 22.1, 68.6)	0.304
Carbohydrate (g)	−5.1 (−24.7, 14.5)	0.599	8.9 (− 8.8, 26.7)	0.312	9.4 (− 13.3, 32.1)	0.406
Total sugars (g)	1.9 (−23.3, 27.2)	0.878	0.5 (−19.7, 20.7)	0.961	−1.7 (−28.5, 25.2)	0.900
Dietary fiber (g)	−13.2 (−81.5, 55.1)	0.697	8.8 (− 176.5, 194)	0.924	38.7 (− 220, 297.5)	0.762
Total fat (g)	11.1 (−38, 60.2)	0.648	−36.2 (− 85.7, 13.3)	0.146	−58 (− 115.2, − 0.7)	0.048^*^
Total saturated fatty acids (g)	1.3 (− 93.9, 96.5)	0.978	− 69.2 (− 180.3, 42)	0.214	− 132.5 (− 300.9, 35.9)	0.119
Cholesterol (mg)	2.2 (−2.6, 6.9)	0.359	−2.7 (−8.2, 2.8)	0.325	0.9 (− 5.7, 7.5)	0.782

^a^ In each multivariate model, race, sex, body weight, body height, family income, physical activity (whenever available), soft drink availability, and total energy intake were adjusted

Results were presented as beta (B) values along with corresponding 95% confidence intervals (CI) and *p*-values

^*^ indicated significance (*p* < 0.05)

**Table 3:** ^a^ Multivariate analyses of associations between daily micronutrient intakes and femoral neck BMD in three age groups

	Ages 8 to 11 (n = 1004)		Ages 12 to 15 (n = 725)		Ages 16 to 19 (n = 669)	
	B × 10^− 5^ (95% CI)	*p*-value	B × 10^− 5^ (95% CI)	*p*-value	B × 10^− 5^ (95% CI)	*p*-value
Daily vitamin intake						
Vitamin D (D2 + D3) (μg)	90.3 (− 69.7, 250.4)	0.259	− 0.9 (− 224.4, 222.6)	0.994	40.1 (− 172.9, 253)	0.704
Vitamin K (μg)	0.1 (− 8.7, 8.8)	0.990	− 1.9 (− 5.9, 2.2)	0.356	−3.5 (− 22.3, 15.3)	0.707
Vitamin C (mg)	−6.1 (− 18.5, 6.2)	0.320	− 0.8 (− 11.8, 10.1)	0.880	2.5 (− 9.5, 14.5)	0.671
Daily minerals intake						
Calcium (mg)	−0.2 (− 1.8, 1.4)	0.809	−1.7 (− 4, 0.6)	0.148	− 0.1 (− 2.5, 2.3)	0.950
Magnesium (mg)	5.4 (−5.2, 16)	0.309	−3.1 (− 15.4, 9.3)	0.615	17.2 (− 2, 36.3)	0.077
Sodium (mg)	0.9 (0.1, 1.6)	0.031^*^	−0.4 (−1.5, 0.6)	0.428	1 (−0.1, 2.2)	0.082

^a^ In each multivariate model, race, sex, body weight, body height, family income, physical activity (whenever available), soft drink availability, and total energy intake were adjusted

Results were presented as beta (B) values along with corresponding 95% confidence intervals (CI) and *p*-values

^*^ indicated significance (*p* < 0.05)

However, subgroup analyses by sex revealed that the associations between daily sodium intake and femoral neck BMD were no longer significant in both male and female participants aged 8 to 11 (Table 4). Instead, it was found that in male participants, daily total cholesterol intake was significantly and positively associated with femoral neck BMD (B = 5.3 × 10^− 5^, *p* = 0.030), but daily calcium intake was significantly and negatively associated with femoral neck BMD (B = − 2 × 10^− 5^, *p* = 0.008) (Table 4). In contrast, in female participants, daily intakes of vitamin D and magnesium were significantly and positively associated with femoral neck BMD (B = 246.8 × 10^− 5^ and 16.3 × 10^− 5^, *p* = 0.017 and 0.033, respectively; Table 4).

**Table 4:** ^a^ Multivariate analyses of associations between dietary nutrient intakes and femoral neck BMD in three age groups stratified by sex

	Ages 8 to 11 (*n* = 1004)		Ages 12 to 15 (*n* = 725)		Ages 16 to 19 (*n* = 669)	
	Male (*n* = 479)	Female (*n* = 525)	Male (*n* = 358)	Female (*n* = 367)	Male (*n* = 326)	Female (*n* = 343)
	B × 10^−5^ (95% CI)	B × 10^− 5^ (95% CI)	B × 10^− 5^ (95% CI)	B × 10^− 5^(95% CI)	B × 10^− 5^ (95% CI)	B × 10^− 5^ (95% CI)
Daily macronutrient intake						
Protein (g)	14.4 (− 40.4, 69.3)	16.8 (− 49.4, 82.9)	−36.5 (− 98.9, 25.9)	32.8 (− 42.3, 107.8)	23.1 (− 28.1, 74.2)	9.4 (− 99.7, 118.5)
Carbohydrate (g)	−6.4 (− 29.8, 16.9)	−4 (− 28.4, 20.5)	19.6 (− 7.3, 46.5)	− 1.2 (− 21, 18.6)	0.8 (− 24.3, 25.8)	17.6 (− 25.7, 60.9)
Total sugars (g)	2.2 (−24.6, 29)	−7.8 (− 35.2, 19.5)	5 (− 23.8, 33.8)	− 1.1 (− 21.8, 19.6)	− 3.5 (− 37.6, 30.6)	1.4 (− 38.5, 41.3)
Dietary fiber (g)	98.7 (−26.3, 223.7)	− 11.8 (− 99.7, 76.1)	−66.7 (− 263.5, 130.1)	11.1 (− 191.8, 214.1)	41.1 (− 339.9, 422.1)	31.7 (− 204.5, 267.9)
Total fat (g)	25.1 (− 40.7, 90.8)	3.7 (− 51.1, 58.5)	− 51.6 (− 137.3, 34.1)	−40.6 (− 97.4, 16.2)	−46.7 (− 122.3, 29)	−47.4 (− 156.5, 61.7)
Total saturated fatty acids (g)	−1.6 (− 106, 102.8)	−37.3 (− 137.9, 63.3)	− 129.1 (− 301.2, 43.1)	3.3 (− 154.4, 161)	− 171.9 (− 381.6, 37.7)	−53.2 (− 287.2, 180.9)
Cholesterol (mg)	5.3 (0.6, 10)^*^	−3.1 (−9.4, 3.2)	−3.6 (− 11, 3.8)	1 (− 4.8, 6.8)	3.1 (− 5.1, 11.3)	−3.5 (− 13.2, 6.3)
Daily vitamin intake						
Vitamin D (D2 + D3) (μg)	− 144.2 (− 348.2, 59.8)	246.8 (48, 445.6)^*^	13.1 (− 317.9, 344.2)	81.1 (− 178.6, 340.8)	− 89 (− 458.8, 280.8)	227.9 (− 120.5, 576.3)
Vitamin K (μg)	7.4 (− 10.6, 25.5)	− 2 (− 11.7, 7.7)	−1.5 (− 6.4, 3.3)	−4.1 (− 12, 3.7)	−12.1 (− 38.7, 14.4)	9.6 (− 5.6, 24.8)
Vitamin C (mg)	−2.5 (− 15, 10)	− 14.7 (− 29.7, 0.3)	− 5.7 (− 20.1, 8.6)	1.9 (− 9.4, 13.1)	6.9 (− 9.8, 23.5)	− 0.9 (− 12.8, 11)
Daily minerals intake						
Calcium (mg)	−2 (−3.4, − 0.6)^*^	0.7 (− 2, 3.4)	− 1.9 (− 5.3, 1.5)	−0.6 (− 3.5, 2.3)	−0.6 (− 4.4, 3.1)	0.6 (−3.1, 4.3)
Magnesium (mg)	−3.8 (− 18.1, 10.6)	16.3 (1.4, 31.1)^*^	− 8.6 (− 25.6, 8.4)	− 0.2 (− 17.2, 16.7)	26.9 (1.8, 51.9)^*^	10.1 (− 10.4, 30.6)
Sodium (mg)	1 (− 0.1, 2.1)	0.4 (− 0.6, 1.4)	−0.4 (− 1.6, 0.8)	0 (− 1.9, 1.9)	2.1 (0.9, 3.4)^*^	− 0.5 (− 2.1, 1)

^a^ In each multivariate model, race, body weight, body height, family income, physical activity (whenever available), soft drink availability, and total energy intakes were adjusted

Results were presented as beta (B) values along with corresponding 95% confidence intervals (CI) and *p*-values

^*^ indicated significance (*p* < 0.05)

#### Ages 12 to 15 years

Univariate analyses did not find significant associations between daily nutrient intakes and femoral neck BMD in participants aged 12 to 15 (Additional file 1: Table S1). After adjusting for race, sex, body weight, body height, family income, soft drink availability, physical activity, and energy, multivariate analyses also revealed that daily intakes of macronutrients and micronutrients were not significantly associated with femoral neck BMD in participants aged 12 to 15 (Tables 2 and 3). Furthermore, as indicated by subgroup analyses by sex (Table 4), daily nutrient intakes were not significantly associated with femoral neck BMD in participants aged 12 to 15, regardless of sex.

#### Ages 16 to 19 years

No significant associations between daily nutrient intakes and femoral neck BMD in participants aged 16 to 19 were demonstrated by univariate analyses (Additional file 1: Table S1).

On the other hand, after adjusting for race, sex, body weight, body height, family income, soft drink availability, physical activity, and energy, multivariate analyses identified a significant and negative association between daily total fat intake and femoral neck BMD in participants aged 16 to 19 (B = − 58 × 10^− 5^, *p* = 0.048; Table 2), but no significant associations between daily micronutrient intakes and femoral neck BMD (Table 3).

Upon stratification by sex, although daily fat intake was not significantly associated with femoral neck BMD in either subgroups, a negative trend of association could be observed (males: B = − 46.7 × 10^− 5^, *p* = 0.218; females: B = − 47.4 × 10^− 5^, *p* = 0.382) (Table 4). In addition, subgroup analyses by sex showed that daily intakes of magnesium and sodium were significantly and positively associated with femoral neck BMD in male participants aged 16 to 19 (B = 26.9 × 10^− 5^ and 2.1 × 10^− 5^, *p* = 0.036 and 0.002, respectively; Table 4). However, no significant associations between daily nutrient intakes and femoral neck BMD were found in female participants (Table 4).

## Discussion

Dietary nutrition intake and lifestyle behaviors may impact the femoral neck BMD and the risk of fracture during childhood and later in life. The current study assessed the potential contribution of macronutrients and micronutrients to BMD in older children and adolescents in a cross-sectional manner. The study found that the associations of specific nutrition-related variables with femoral neck BMD is dependent upon age and sex. Multivariate analysis found that sodium was significantly associated with femoral neck BMD in a positive manner in participants aged between 8 to 11 years, while only daily total fat intake showed a significant and positive association with femoral neck BMD in participants aged 16 to 19 years. Neither of these associations were significant upon subgroup stratification by sex. Instead, daily intakes of calcium and cholesterol were associated with femoral neck BMD in 8- to 11-year-old males significantly, while daily intakes of vitamin D and magnesium were both associated with femoral neck BMD in females of the same age group. In male participants aged 16 to 19 years, the daily intakes of magnesium and sodium were found positively associated with femoral neck BMD with statistical significance, but no nutritional factors were found associated with femoral neck BMD in females of the same group.

The difference between age groups may reflect the interaction of dietary nutrition intake and the age-specific pattern of bone growth [12]. Bone mass is acquired slowly throughout childhood, but increases rapidly with the onset of puberty, as during ages of 8 to 11 years, [18] and bone mineral accretion peaks shortly after peak height gain [12]. For total body bone mineral, the peak bone accretion occurs at about 12.5 years of age in girls and 14 years of age in boys [12]. During 2 years prior and 2 years post this peak in bone accretion, 39% of total bone mineral is acquired [12]. Prior studies suggest that during this time of rapid accretion the skeleton may be most at risk of dietary insufficiencies [18]. Conditions related to dietary deficiencies that may manifest during childhood include rickets, osteomalacia, low trauma fractures, and stunting [12, 19]. Meanwhile, intakes of calcium, fruit, and vegetables during adolescent years have been shown beneficial for BMD of the whole body or forearm [20–22], which is different to the primary outcome of interest in the current study. Nevertheless, body weight was considered as the main factor that could predict with BMD in the femoral neck of both males and females at late teenage-hood and young adulthood [23].

As pointed out by a NHANES study [24], femoral neck BMD was 5–13% higher in males than females, except in those age 12–15. Hence, it is reason to assume that the association between nutritional factors and femoral neck BMD may be sex- and age-dependent. A longitudinal cohort study conducted in Northern Ireland found that daily intakes of calcium, phosphorus, and vitamin D were associated femoral neck BMD in females but not in males [23]. These sex-specific attributes were also reported by a Korean cross-sectional study, which found that vitamin D deficiency was associated with femoral neck BMD in only female adolescents [25]. These sex-specific differences may be attributed to differential nutrient requirements for bone growth and development through childhood and adolescence between males and females. Differential skeletal maturation in relation to sex and age has been supported by a Korean population-based study, which observed that BMD of the whole body increases with age, peaking at ages 17–20 years in females and at ages 20–23 in males [6]. Furthermore, estrogens and androgens, sex hormones responsible for sexual dimorphism, differentially regulate the growth and maintenance of bones and muscles between males and females [26].

The roles of vitamin D, magnesium, and fat intakes in BMD have been demonstrated by several groups, although with different outcome or age group designs compared to the current study. Zhou et al. (2013) reported that higher vitamin D intake was associated with higher femoral neck BMD in young men and women aged 16–24 years in the Canadian Multicentre Osteoporosis Study [13]. Despite this, a randomized intervention study with daily vitamin supplements in children of 6–8 years over the winter months did not find any significant improvements in BMD of femoral neck, total body or total body less head after 3 years [27]. Dietary magnesium intakes from milk consumption, but not from other foods, was found to associate with lumbar bone BMD in female adolescents [28]. Another interventional study using magnesium oxide supplements found that healthy 8- to 14-year-old girls benefited from this supplement, especially in hip BMD [29]. These findings may not directly correlate with our findings as the age groups or BMD measurements differed slightly from the current study, but they highlight the scientific interest and often times conflicting results of how vitamin D and magnesium may potentially contribute to bone mass accrual. The current assumption on the potentially negative role of high daily fat intake in BMD is more consistent [30, 31], and this is reflected by our findings in the adolescent group (16- to 19-year-olds). However, most of the available studies on daily fat intakes were performed in adults or even postmenopausal women, and a translation to adolescents requires further investigation.

The positive association of daily sodium intakes with femoral bone BMD found in the current study is, nevertheless, paradoxical. Most studies, as exemplified by a meta-analysis, found that excess sodium intake was unfavorable for bone quality in adults [32]. The systematic review performed by the National Osteoporosis Foundation for peak bone mass development found that available data did not favor dietary sodium intakes [12, 33]. It should be noted that the beta coefficients reported for sodium was rather small in the current report, and therefore less likely to be clinically relevant. This finding may be a result of strict exclusion criteria (i.e. extreme obesity and implausible calorie intake) and statistical adjustments (e.g. anthropometric measurements, total energy intake, lifestyle factors, and etc.) applied in order to eliminate potential bias but may have offsetted the findings. Furthermore, the small value reported herein may be due to a combined effect of the femoral bone BMD being very small (less than 1 g/cm^2^) and the sodium intake value being large (in thousands of mg).

The current study possesses some limitations. First of all, this study analyzed the data from NHANES that are comprehensive and nationally representative of the United States. As a result, the current findings are likely to represent the general U.S. population, but may not be translatable to other geographic regions. In addition, the NHANES dataset is cross-sectional making it not possible to infer causality. Growth and mineralization differ across the bones of the body. The findings of this study are relevant to the femur only. While, to understand the developmental impact of daily nutrient intakes on bone growth and development throughout childhood and adolescence, further studies are needed to evaluate the relationship of our findings to other parts of the skeletal structure, including the lumbar spine [24].

Moreover, the current study design may possess some intrinsic limitations; therefore, the results should be interpreted with caution. In the present study, 789 participants with extreme obesity were excluded, since those obese persons often associated with abnormal eating behaviors [34]. Furthermore, because of inclusion and exclusion criteria, the study population of the present study consisted only 2398 participants. After stratified by age and sex, the sizes of the individual subpopulations became smaller. Such small sample size may dampen the representativeness of the study subpopulations, which may be at least in part responsible for the observed negative association between calcium intake and femoral neck BMD in males aged 8–11. In addition, the small range of measured femoral neck BMD may also contribute to the above-mentioned negative association in male children to some degree. Furthermore, a significant and negative association between daily total fat intake and femoral neck BMD with a *p*-value of 0.048 was observed in participants aged 16 to 19 in the present study. However, although this p-value is less than the default p-value threshold for statistical significance, 0.05, such near-threshold p-value makes interpretation difficult [35]. Due to marginal significance, further investigations with large sample size are required to confirm the current findings.

## Conclusion

Current guidelines do not specify age-specific suggestions for daily intake of nutrients that impact bone growth and mineralization. Although not conclusive, the current findings might suggest age- and sex-specific daily nutritional requirements for bone growth and development during childhood and through puberty. Additional studies are necessary to further analyze the contribution of specific dietary factors with bone growth and development in children through young adults.

## Supplementary information

**Additional file 1: Table S1.** Univariate analyses of associations between dietary nutrient intakes and femoral neck BMD in three age groups.

## Data Availability

The data that support the findings of this study are available from the corresponding author, upon reasonable request.

## Abbreviations

BMD: Bone mineral density
NHANES: National Health and Nutrition Examination Survey
SE: Standard errors
