# Supplementary material for: Nutritional factors associated with femoral neck bone mineral density in children and adolescents
Source: BMC Musculoskelet Disord. 2019 Nov 7;20:520. doi: 10.1186/s12891-019-2901-9 (PMC6839089; doi:10.1186/s12891-019-2901-9)
Supplement: Supplementary file 1 — Additional file 1: Table S1. Univariate analyses of associations between dietary nutrient intakes and femoral neck BMD in three age groups. [file 12891_2019_2901_MOESM1_ESM.docx]

Additional file 1: **Table S1.** Univariate analyses of associations between dietary nutrient intakes and femoral neck BMD in three age groups.

|  | Ages 8 to 11 (n=1,004) | |  | Ages 12 to 15 (n=725) | |  | Ages 16 to 19 (n=669) | |
| --- | --- | --- | --- | --- | --- | --- | --- | --- |
|  | B x 10^-5^ (95% CI) | p-value |  | B x 10^-5^ (95% CI) | p-value |  | B x 10^-5^ (95% CI) | p-value |
| **Daily total nutrition intake** |  |  |  |  |  |  |  |  |
| Energy (kcal) | 1.2 (0.2, 2.2) | 0.018* |  | 0.1 (-1.7, 1.8) | 0.924 |  | 0.7 (-1.7, 3.2) | 0.534 |
| Protein (g) | 30.4 (0.2, 60.6) | 0.049* |  | -10.3 (-46.6, 26.1) | 0.568 |  | 17.4 (-22.4, 57.2) | 0.381 |
| Carbohydrate (g) | 5 (-2.9, 12.9) | 0.207 |  | 2.8 (-7.1, 12.8) | 0.566 |  | 8.6 (-6.1, 23.3) | 0.241 |
| Total sugars (g) | 7.7 (-8.8, 24.3) | 0.348 |  | 2.1 (0, 0) | 0.742 |  | 6.2 (-12.4, 24.8) | 0.503 |
| Dietary fiber (g) | 35.1 (-29.7, 100) | 0.278 |  | -1.7 (-146.6, 143.2) | 0.981 |  | 26.2 (-215, 267.5) | 0.826 |
| Total fat (g) | 24.7 (2.7, 46.7) | 0.029* |  | -10.8 (-42.4, 20.8) | 0.490 |  | -8.1 (-54.6, 38.4) | 0.724 |
| Total saturated fatty acids (g) | 43 (-3.7, 89.8) | 0.070 |  | -27.1 (-113.2, 59) | 0.526 |  | -29.8 (-172.7, 113) | 0.673 |
| Cholesterol (mg) | 3.7 (-0.4, 7.8) | 0.075 |  | -2.2 (-7.5, 3.2) | 0.415 |  | 0.5 (-5.2, 6.1) | 0.869 |
| **Daily vitamin intake** |  |  |  |  |  |  |  |  |
| Vitamin D (D2 + D3) (μg) | 134.8 (-7.5, 277.2) | 0.063 |  | -11.4 (-222.7, 199.9) | 0.913 |  | 22.4 (-179.7, 224.5) | 0.823 |
| Vitamin K (μg) | 6.2 (-2.3, 14.6) | 0.147 |  | -2.7 (-7.1, 1.7) | 0.223 |  | -2.4 (-23, 18.2) | 0.814 |
| Vitamin C (mg) | -0.5 (-13.2, 12.1) | 0.935 |  | 0.4 (-10.6, 11.4) | 0.939 |  | 8.4 (-4.6, 21.4) | 0.198 |
| **Daily minerals intake** |  |  |  |  |  |  |  |  |
| Calcium (mg) | 0.6 (-0.7, 1.8) | 0.349 |  | -1 (-3.2, 1.2) | 0.373 |  | 0.4 (-1.6, 2.5) | 0.680 |
| Magnesium (mg) | 8 (0.4, 15.7) | 0.040* |  | -2 (-14, 10) | 0.732 |  | 12.4 (-2.1, 27) | 0.092 |
| Sodium (mg) | 0.9 (0.4, 1.4) | <.001* |  | -0.3 (-1, 0.5) | 0.487 |  | 0.7 (-0.2, 1.7) | 0.130 |

Results were presented as beta (B) values along with corresponding 95% confidence intervals (CI) and p-values.

* indicated significance (p<0.05).
